# Clinical implication of ticagrelor monotherapy in patients with small vessel coronary artery disease: results from the TICO randomized trial

**DOI:** 10.3389/fcvm.2023.1237826

**Published:** 2023-08-08

**Authors:** Jae Young Cho, Donghyeon Joo, Kyeong Ho Yun, Byeong-Keuk Kim, Myeong-Ki Hong, Yangsoo Jang, Seok Kyu Oh

**Affiliations:** ^1^ Departments of Cardiovascular Medicine, Regional Cardiocerebrovascular Center, Wonkwang University Hospital, Iksan, Republic of Korea; ^2^ Severance Cardiovascular Hospital, Yonsei University College of Medicine, Seoul, Republic of Korea; ^3^ Division of Cardiology, Department of Internal Medicine, CHA Bundang Medical Center, CHA University, Seongnam, Republic of Korea

**Keywords:** coronary artery disease, drug-eluting stent, dual anti-platelet therapy, ticagrelor, prognosis

## Abstract

**Background:**

The aim of this study was to evaluate the efficacy and safety of ticagrelor monotherapy in patients with small vessel disease compared with ticagrelor-based DAPT within the Ticagrelor Monotherapy after 3 Months in the Patients Treated with New Generation Sirolimus Eluting Stent for Acute Coronary Syndrome (TICO) trial population.

**Methods:**

Reference vessel diameter ≤2.5 mm was considered as small vessel disease. We conducted a comparison of the incidence of target lesion failure (TLF) and Bleeding Academic Research Consortium (BARC) type 3 or 5 bleeding. TLF was defined as a composite of cardiac death, target lesion myocardial infarction, stent thrombosis, and target lesion revascularization.

**Results:**

652 patients among 3,056 TICO population (21.3%) had small vessel disease. Patients with small vessel disease showed a higher rate of TLF compared to those without small vessel disease (2.9% vs. 1.0%, log-rank *p* < 0.001). The presence of small vessel disease emerged as an independent predictor for 1-year TLF (HR 2.84, 95% CI 1.54–5.25), while it did not show a significant association with bleeding complications. The 12-month TLF rate was 1.6% for ticagrelor monotherapy after 3-month DAPT, and 4.2% for ticagrelor-based 12-month DAPT (*p* = 0.059) in patients with small vessel disease (HR 0.38, 95% CI 0.14–1.04, *p* for interaction = 0.261). The incidence of BARC type 3 or 5 bleeding rate 2.5% for ticagrelor monotherapy after 3-month DAPT, and 5.6% for ticagrelor-based 12-month DAPT (*p* = 0.052) in patients with small vessel disease (HR 0.44, 95% CI 0.19–1.01, *p* for interaction = 0.322). In the 3-month landmark analysis, ticagrelor monotherapy significantly reduced BARC type 3 or 5 bleeding in patients with small vessel disease (HR 0.09, 95% CI 0.01–0.69, log-rank *p* = 0.005) while demonstrating a similar incidence of TLF compared to ticagrelor based 12-month DAPT during the 3–12 months period.

**Conclusions:**

There are no significant interactions between the antiplatelet strategy regarding the 12-month incidence of ischemic and bleeding complications. Ticagrelor monotherapy demonstrated a reduction in bleeding complications after a 3-month period of DAPT without increasing the rate of TLF, when compared to ticagrelor-based 12-month DAPT, specifically in patients with small vessel disease.

**Clinical Trial Registration**: www.ClinicalTrials.gov, identifier, NCT02494895.

## Introduction

Despite the development of newer generation stents and techniques, small-vessel coronary artery disease continues to pose challenges for percutaneous coronary intervention (PCI) (1, 2). Small vessel disease is associated with a higher incidence of restenosis and stent thrombosis, leading to target lesion failure (TLF) (1–3). Several studies have aimed to identify the most effective device for patients with small vessel disease, yet the antiplatelet regimen for these patients remains poorly defined.

Ticagrelor Monotherapy after 3 Months in the Patients Treated with New Generation Sirolimus Eluting Stent for Acute Coronary Syndrome (TICO) trial demonstrated that ticagrelor monotherapy, following 3 months of dual antiplatelet therapy (DAPT), reduced the risk of bleeding without increasing the risk of ischemic complications when compared to 12 months of DAPT with aspirin and ticagrelor (4). Nevertheless, further studies are needed to assess specific subgroups that exhibit a high risk of ischemic or bleeding events. The objective of this study was to evaluate the efficacy and safety of ticagrelor monotherapy in patients with small vessel disease compared with ticagrelor-based DAPT within the TICO trial population.

## Methods

### Study population

This is a sub-study of the TICO randomized trial (clinicaltrials.gov identifier: NCT02494895), and the study design has been previously published (5). In summary, a total of 3,056 patients with acute coronary syndrome were randomly assigned to either ticagrelor monotherapy after a 3-month DPAT or ticagrelor-based 12-month DAPT with aspirin and ticagrelor. The patients underwent successful PCI with ultrathin bioresorbable polymer sirolimus-eluting stents (Orsiro, Biotronik AG, Berlin, Germany). Key exclusion criteria included patients with a history of hemorrhagic stroke, internal bleeding within the past 6 weeks, traumatic brain injury or brain surgery within the past 6 months, the need for oral anticoagulation therapy, and anemia (hemoglobin ≤8 g/dl). For this sub-study, the patients were categorized into those with small vessel disease and those with non-small vessel disease based on a reference vessel size of 2.5 mm.

### Angiographic analysis

Angiographic analysis was conducted for all procedures, ensuring complete data collection. Quantitative coronary analysis (QCA) of the angiographic images was performed using offline software (CAAS system, Pie Medical Imaging, Maastricht, the Netherlands) at a central core laboratory (Cardiovascular Research Center, Seoul, Republic of Korea) by specialized technicians who were blinded to the contents and purpose of this study. The reference vessel diameter was determined as an average of the proximal and distal diameters. For the purpose of this study. small vessel coronary artery disease was defined as a minimal reference diameter ≤2.5 mm (6, 7).

### Endpoint

The outcomes of the present study focused on evaluating ischemic and bleeding complications occurring within 12 months after PCI. The primary ischemic outcome was TLF, defined as a composite endpoint encompassing cardiac death, target vessel myocardial infarction, stent thrombosis, and target lesion revascularization. Additionally, all-cause death, ischemic stroke, and any revascularization were analyzed as additional ischemic complications in accordance with the criteria established by the Academic Research Consortium (8). Bleeding complications were assessed based on the Bleeding Academic Research Consortium (BARC) definition, specifically examining BARC type 3 or 5 bleeding events (9).

### Statistical analyses

Baseline characteristics were compared between patients with small vessel disease and those with non-small vessel disease using independent *t*-test or the Mann–Whitney *U* test for continuous variables and using a *χ*^2^ test or Fisher’s exact test for categorical variables. Cumulative incidences of ischemic and bleeding complications were determined using Kaplan-Meier estimates and assessed through the log-rank test. Cox proportional hazard model was constructed to identify contributing factors to target vessel failure and bleeding complications, and the results were reported as hazard ratios (HR) with corresponding 95% confidence intervals (CI). The analysis adjusted for several covariates including age, gender, body mass index, hypertension, diabetes to assess the impact of small vessel disease and the antiplatelet strategy on TLF and bleeding complications. Treatment effects of the antiplatelet strategy were estimated based on the presence or absence of small vessel disease, with interaction terms included in the Cox proportional hazard model. A 3-month landmark analysis was performed, excluding patients who experienced ischemic or bleeding complications within this period, as all patients received the same treatment during the first 3 months. The statistical analyses were conducted on the intension-to-treat cohort using R version 4.0.2. (The R Foundation for Statistical Computing, Vienna, Austria). All *p*-values are two-sided, and statistical significance was defined as a *p*-value below 0.05.

## Results

**Figure 1:**
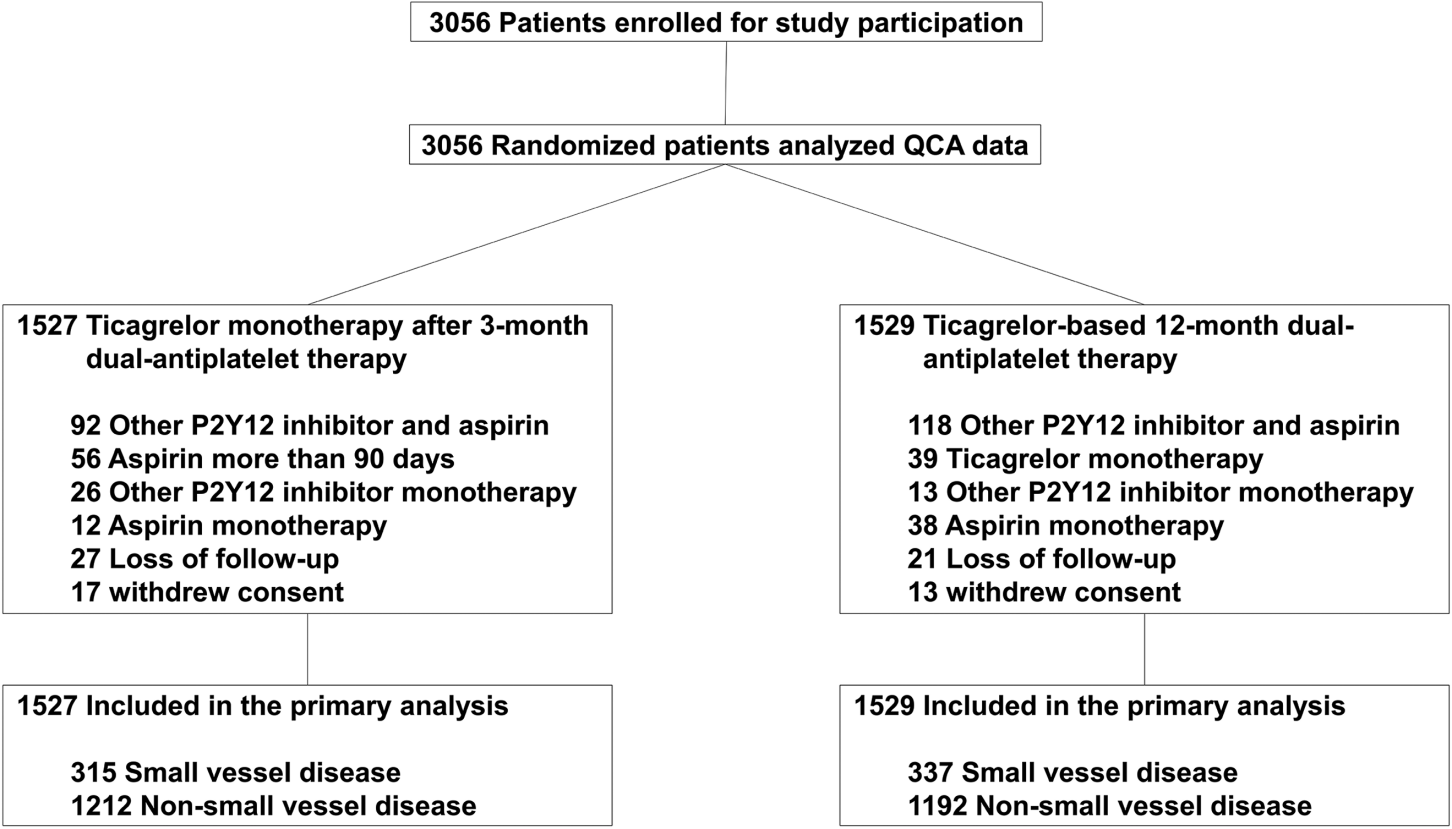
Participant flow in the present study.

Among the 3,056 patients enrolled in the TICO trial, 652 patients (21.3%) had small vessel disease. The incidence of small vessel disease was similar between the two antiplatelet treatment strategies in the sub-analysis (Figure 1). Table 1 presented the baseline characteristics of the patients. Patients with small vessel disease were older, and had a higher incidence of female gender, hypertension, diabetes mellitus, and multi-vessel disease compared to those with non-small vessel disease. All patients received ultrathin bioresorbable polymer sirolimus-eluting stents (Orsiro stent) according to the study protocol. Smaller and longer stents were used in patients with small vessel disease.

**Table 1:** Baseline clinical characteristics.

	Small vessel disease	Non-small vessel disease	*p* value
	(*n* = 652)	(*n* = 2,404)	
Age (years)	62.3 ± 10.6	60.6 ± 10.8	<0.001
Female (%)	180 (27.6)	448 (18.6)	<0.001
Body mass index (kg/m^2^)	24.8 ± 3.3	25.0 ± 3.2	0.276
Hypertension (%)	359 (55.1)	1,182 (49.2)	0.009
Diabetes mellitus (%)	208 (31.9)	627 (26.1)	0.004
Dyslipidemia (%)	402 (61.7)	1,444 (60.1)	0.489
Current smoker (%)	225 (34.5)	917 (38.1)	0.098
Previous stroke (%)	36 (5.5)	90 (3.7)	0.056
Myocardial infarction (%)	433 (66.4)	1,697 (70.6)	0.044
Ejection fraction (%)	54.1 ± 12.7	54.7 ± 11.8	0.293
Hemoglobin (g/dl)	14.1 ± 1.8	14.3 ± 1.7	0.003
eGFR (ml/min/1.73 m^2^)	77.5 ± 26.6	76.5 ± 22.5	0.423
12-month DPAT group	337 (51.7)	1,192 (49.6)	0.364
Culprit lesion (%)			<0.001
Left main	7 (1.1)	73 (3.0)	
Left anterior descending	345 (52.9)	1,183 (49.2)	
Left circumflex	178 (27.3)	332 (13.8)	
Right coronary artery	122 (18.7)	816 (33.9)	
Multi-vessel disease (%)	386 (59.2)	1,317 (54.8)	0.049
Femoral approach (%)	286 (43.9)	1,072 (44.6)	0.774
Stent number per patient	1.41 ± 0.70	1.36 ± 0.66	0.069
Minimal stent diameter (mm)	2.70 ± 0.24	3.26 ± 0.41	<0.001
Total stent length (mm)	37.7 ± 22.1	34.0 ± 20.1	<0.001
Reference diameter (mm)	2.32 ± 0.16	3.07 ± 0.40	<0.001
Minimal luminal diameter (mm)	0.36 ± 0.36	0.51 ± 0.49	<0.001
Pre-procedural diameter stenosis (%)	84.7 ± 15.1	83.5 ± 15.4	0.060
Lesion length (mm)	26.2 ± 14.0	23.5 ± 12.6	<0.001
Post-procedural diameter stenosis (%)	14.6 ± 7.9	14.9 ± 7.6	0.414
Discharge medication			
ACEI/ARB (%)	440 (67.5)	1,579 (65.7)	0.415
Beta blocker (%)	431 (66.1)	1,585 (65.9)	0.971
CCB (%)	96 (14.7)	301 (12.5)	0.156
Statin (%)	638 (97.9)	2,355 (98.0)	0.985

eGFR, estimated glomerular filtration rate; DAPT, dual antiplatelet therapy; ACEI, angiotensin converting enzyme inhibitor; ARB, angiotensin receptor blocker; CCB, calcium channel blocker.

**Figure 2:**
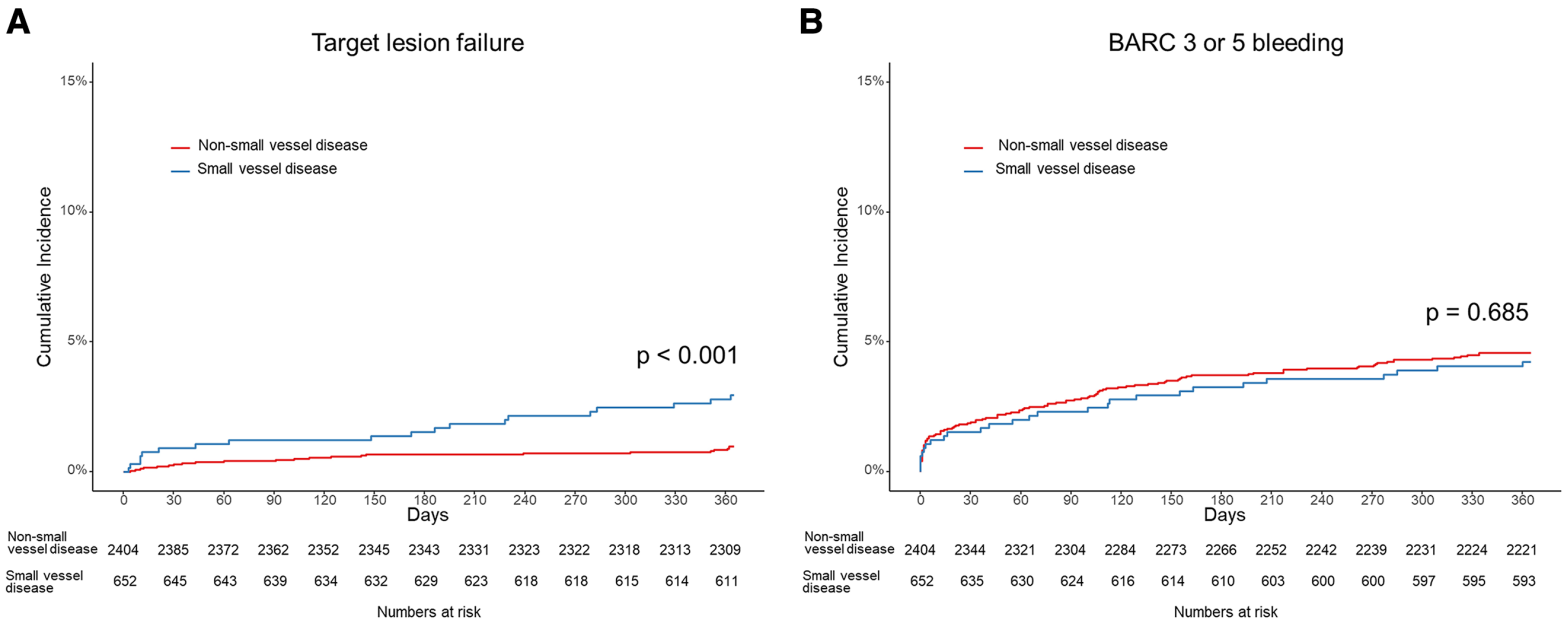
Cumulative incidence of (**A**) target lesion failure including cardiac death, target vessel myocardial infarction, stent thrombosis and target lesion revascularization, and (**B**) bleeding academic research consortium (BARC) definition 3 or 5 bleeding in patients with small vessel disease and those with non-small vessel disease.

The patients with small vessel disease showed a higher TLF rate than those with non-small vessel disease (2.9% vs. 1.0%, log-rank *p* < 0.001) (Figure 2). Specifically, the patients with small vessel disease experienced a higher incidence of cardiac death and stent thrombosis (Table 2). In contrast, bleeding complications, including each component of the BARC definition and the total event, showed a similar incidence between the patients with small vessel disease and those without small vessel disease. The presence of small vessel disease was an independent predictor for 1-year TLF (HR 2.84, 95% CI 1.54–5.25) (Table 3). Regarding bleeding complications, age (HR 1.55, 95% CI 1.08–2.22), renal dysfunction (HR 1.87, 95% CI 1.30–2.70), and antiplatelet regimen (HR 0.65, 95% CI 0.46–0.92) were independent predictors, while small vessel disease was not.

**Table 2:** 1-year outcomes in patients with small vessel disease and without small vessel disease.

	Small vessel disease	Non-small vessel disease	HR (95% CI)	*p* value
	(*n* = 652)	(*n* = 2,404)		
Ischemic events (%)				
All cause death	12 (1.8)	27 (1.1)	1.65 (0.84, 3.26)	0.149
Cardiac death	10 (1.5)	9 (0.4)	4.11 (1.67, 10.12)	0.002
Myocardial infarction	7 (1.1)	10 (0.4)	2.61 (0.99, 6.85)	0.052
Stent thrombosis	5 (0.8)	5 (0.2)	3.70 (1.07, 12.79)	0.039
Target lesion revascularization	3 (0.5)	7 (0.3)	1.60 (0.41, 6.20)	0.495
Any revascularization	8 (1.2)	26 (1.1)	1.15 (0.52, 2.54)	0.729
Target lesion failure	19 (2.9)	23 (1.0)	3.07 (1.67, 5.65)	<0.001
MACE^a^	34 (5.2)	72 (3.0)	1.77 (1.18, 2.66)	0.006
Bleeding events (%)				
BARC 3A	10 (1.5)	50 (2.1)	0.74 (0.37, 1.46)	0.383
BARC 3B	15 (2.3)	53 (2.2)	1.05 (0.59, 1.86)	0.867
BARC 3C	2 (0.3)	4 (0.2)	1.86 (0.34, 10.14)	0.475
BARC 5	0 (0.0)	2 (0.1)		0.619
BARC 3 or 5	27 (4.1)	109 (4.5)	0.92 (0.60, 1.40)	0.685

HR, hazard ratio; CI, confidence interval; BARC, Bleeding Academic Research Consortium.

^a^ Major adverse cardiac and cerebrovascular events indicated composite of death, myocardial infarction, stent thrombosis, stroke, and target vessel revascularization.

**Table 3:** Predictors of target lesion failure and bleeding events in ticagrelor treated patients.

	Univariate analysis		Multivariate analysis	
	HR (95% CI)	*p*	HR (95% CI)	*p*
Target lesion failure				
Small vessel disease	3.07 (1.67, 5.65)	<0.001	2.84 (1.54, 5.25)	<0.001
Ticagrelor monotherapy	0.56 (0.30, 1.05)	0.069	0.58 (0.31, 1.08)	0.086
Age >65 years	2.34 (1.27, 4.34)	0.007	1.79 (0.94, 3.44)	0.078
Female gender	1.37 (0.69, 2.73)	0.366	0.82 (0.40, 1.70)	0.600
Diabetes mellitus	3.6 (1.95, 6.63)	<0.001	2.92 (1.56, 5.46)	<0.001
eGFR <60	2.49 (1.34, 4.64)	0.004	1.83 (0.96, 3.49)	0.068
Bleeding complications				
Small vessel disease	0.92 (0.60, 1.40)	0.685	0.83 (0.54, 1.27)	0.395
Ticagrelor monotherapy	0.64 (0.45, 0.90)	0.011	0.65 (0.46, 0.92)	0.016
Age >65 years	1.98 (1.41, 2.78)	<0.001	1.55 (1.08, 2.22)	0.018
Female gender	1.82 (1.27, 2.62)	0.001	1.46 (1.00, 2.15)	0.051
Diabetes mellitus	1.73 (1.22, 2.44)	0.002	1.40 (0.98, 2.00)	0.066
eGFR <60	2.31 (1.63, 3.28)	<0.001	1.87 (1.30, 2.70)	<0.001

HR, hazard ratio; CI, confidence interval; eGFR, estimated glomerular filtration rate.

The antiplatelet strategy was not associated with ischemic and bleeding complications, regardless of the presence or absence of small vessel disease, after adjusting covariates (Table 4). Additionally, no significant group interactions were observed. In patients with small vessel disease, there are no significant difference in the incidence of ischemic and bleeding endpoints between the two treatment strategies. The TLF rate was 1.6% in the ticagrelor monotherapy after 3-month DAPT group and 4.2% in the ticagrelor-based 12-month DAPT group (*p* = 0.059) (HR 0.38, 95% CI 0.14–1.04, *p* for interaction = 0.261). The BARC 3 or 5 bleeding rate was 2.5% in the ticagrelor monotherapy after 3-month DAPT group and 5.6% in the ticagrelor-based 12-month DAPT group (*p* = 0.052) (HR 0.44, 95% CI 0.19–1.01, *p* for interaction = 0.322). Similar findings were observed in patients with non-small vessel disease.

**Table 4:** Adjusted hazard ratio according to treatment group in patients with and without SVD.

	Ticagrelor-based DAPT	Ticagelor mono therapy	HR (95% CI)	*p* value	*p* value (interaction)
Death					
SVD	8/337 (2.4)	4/315 (1.3)	0.53 (0.16, 1.74)	0.292	0.496
Non-SVD	15/1,192 (1.3)	12/1,212 (1.0)	0.85 (0.40, 1.81)	0.666	
Cardiac death					
SVD	7/337 (2.1)	3/315 (1.0)	0.45 (0.12, 1.73)	0.242	0.506
Non-SVD	5/1,192 (0.4)	4/1,212 (0.3)	0.84 (0.23, 3.13)	0.796	
Myocardial infarction					
SVD	5/337 (1.5)	2/315 (0.6)	0.42 (0.08, 2.18)	0.302	0.663
Non-SVD	6/1,192 (0.5)	4/1,212 (0.3)	0.68 (0.19, 2.41)	0.549	
Stent thrombosis					
SVD	2/337 (0.6)	3/315 (1.0)	1.49 (0.25, 8.99)	0.662	0.974
Non-SVD	2/1,192 (0.2)	3/1,212 (0.2)	1.56 (0.26, 9.37)	0.625	
Ischemic stroke					
SVD	6/337 (1.8)	4/315 (1.3)	0.70 (0.20, 2.47)	0.574	0.702
Non-SVD	8/1,192 (0.7)	4/1,212 (0.3)	0.50 (0.15, 1.66)	0.259	
TLR					
SVD	2/337 (0.6)	1/315 (0.3)	0.53 (0.05, 5.81)	0.601	0.793
Non-SVD	4/1,192 (0.3)	3/1,212 (0.2)	0.77 (0.17, 3.46)	0.738	
TLF					
SVD	14/337 (4.2)	5/315 (1.6)	0.38 (0.14, 1.04)	0.059	0.261
Non-SVD	13/1,192 (1.1)	10/1,212 (0.8)	0.80 (0.35, 1.81)	0.586	
Any revascularization					
SVD	5/337 (1.5)	3/315 (1.0)	0.63 (0.15, 2.65)	0.532	0.349
Non-SVD	11/1,192 (0.9)	15/1,212 (1.2)	1.38 (0.63, 3.00)	0.419	
Any ischemic events					
SVD	23/337 (6.8)	11/315 (3.5)	0.50 (0.24, 1.02)	0.058	0.163
Non-SVD	38/1,192 (3.2)	34/1,212 (2.8)	0.92 (0.57, 1.46)	0.710	
BARC 3A bleeding					
SVD	5/337 (1.5)	5/315 (1.6)	1.06 (0.31, 3.68)	0.922	0.502
Non-SVD	30/1,192 (2.5)	20/1,212 (1.7)	0.67 (0.31, 1.18)	0.164	
BARC 3B bleeding					
SVD	13/337 (3.9)	2/315 (0.6)	0.16 (0.04, 0.71)	0.016	0.053
Non-SVD	30/1,192 (2.5)	23/1,212 (1.9)	0.77 (0.45, 1.33)	0.344	
BARC 3C bleeding					
SVD	1/337 (0.3)	1/315 (0.3)	1.06 (0.07, 16.93)	0.969	0.960
Non-SVD	2/1,192 (0.2)	2/1,212 (0.2)	0.95 (0.13, 6.77)	0.960	
BARC 5 bleeding					
SVD	0/337 (0.0)	0/315 (0.0)			1.000
Non-SVD	2/1,192 (0.2)	0/1,212 (0.0)		0.999	
BARC 3 or 5 bleeding					
SVD	19/337 (5.6)	8/315 (2.5)	0.44 (0.19, 1.01)	0.052	0.322
Non-SVD	64/1,192 (5.4)	45/1,212 (3.7)	0.70 (0.48, 1.03)	0.068	

DAPT, dual antiplatelet therapy; SVD, small vessel disease; TLR, target lesion revascularization; TLF, target lesion failure; BARC, bleeding academic research consortium.

**Figure 3:**
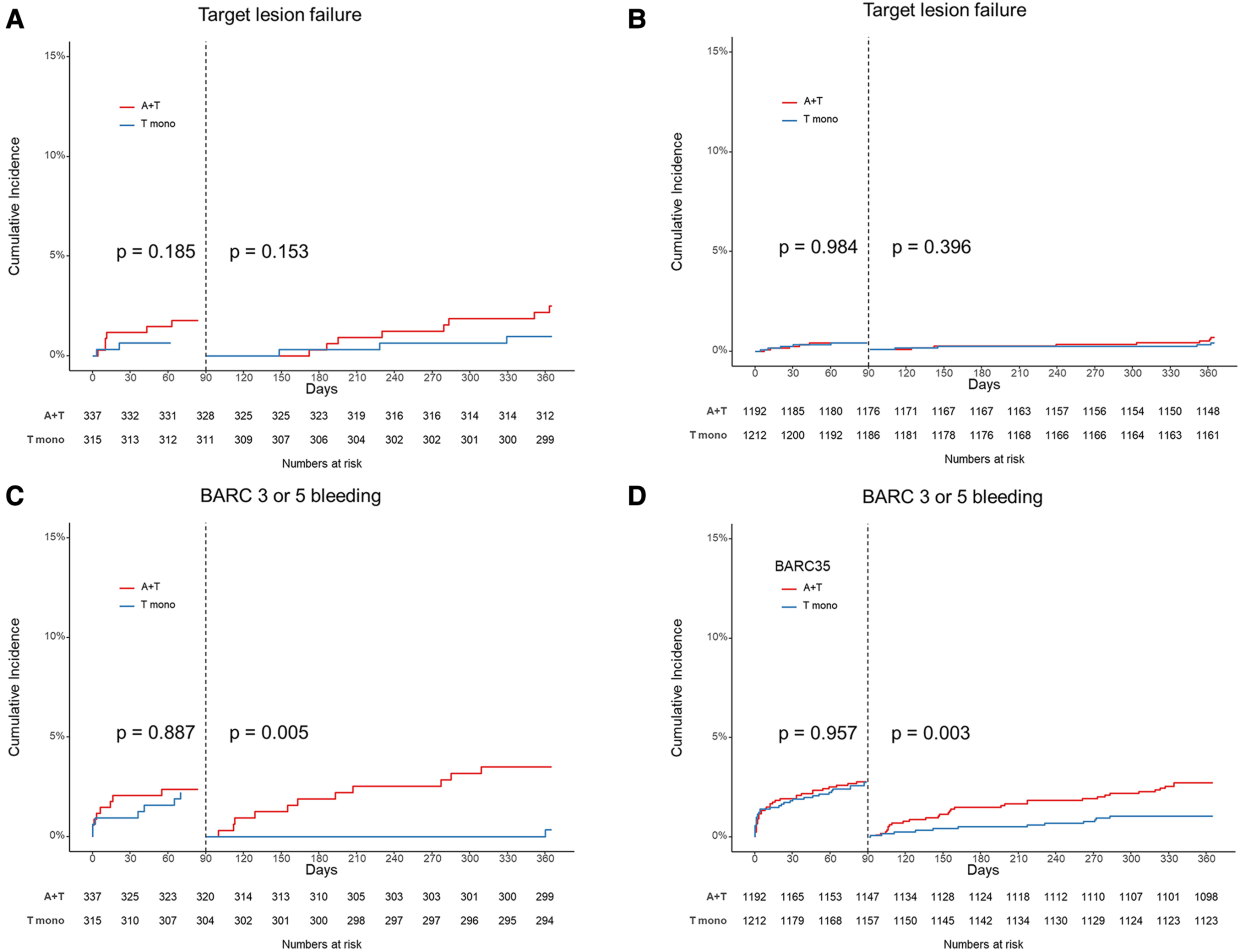
3-month landmark analysis. Target lesion failure in patients with small vessel disease (**A**) and patients with non-small vessel disease (**B**). Bleeding Academic Research Consortium (BARC) definition 3 or 5 bleeding in patients with small vessel disease (**C**) and patients with non-small vessel disease (**D**). A + T indicated ticagrelor based 12-month dual antiplatelet therapy, and T mono indicated ticagrelor monotherapy after 3-month dual antiplatelet therapy.

The 3-month landmark analysis revealed that regardless of the presence or absence small vessel disease, there was a similar incidence of ischemic complications between the two treatment strategies during the 3–12 months period (Figures 3A,B). Between 3 and 12 months, the hazard ratio of TLF for ticagrelor monotherapy was 0.42 (95% CI 0.11–1.60) in patients with small vessel disease and 0.68 (95% CI 0.22–2.08) in patients with non-small vessel disease. However, ticagrelor monotherapy significantly reduced bleeding complications in the 3-month landmark analysis for both patients with small vessel disease (HR 0.09, 95% CI 0.01–0.69, log-rank *p* = 0.005) and those with non-small vessel disease (HR 0.40, 95% CI 0.20–0.77, log-rank *p* = 0.003) (Figures 3C,D).

## Discussion

In this sub-analysis of the TICO trial, several key findings were observed: (1) small vessel disease was independently associated with a higher risk of 1-year TLF in patients treated with Orsiro stents, (2) ticagrelor-based 12-month DAPT was not significantly associated with a reduced TLF rate in patients with small vessel disease compared to ticagrelor monotherapy after 3 months of DAPT, (3) ticagrelor monotherapy after the 3-month DAPT was significantly associated with a reduced rate of BARC 3 or 5 bleeding in patients with small vessel disease without increasing the risk of ischemic events, and (4) no significant interactions were founded between the antiplatelet strategy and the presence or absence of small vessel disease regarding the occurrence of ischemic and bleeding complications.

Several definitions have been proposed for small vessel disease, including an angiographic reference vessel diameter of equal to or less than 2.75 mm or 2.5 mm (6, 7, 10). Despite variations in these definitions, small vessel disease has consistently been associated with an increased risk of adverse events, including restenosis and thrombosis (1, 3, 11, 12). However, newer generation drug-eluting stents with thinner struts and more biocompatible or biodegradable polymers have shown improved clinical outcomes compared to early generation stents. A subgroup analysis of the XIENCE V USA study, which included 2,853 patients treated with XIENCE V everolimus eluting-stent, found no significant difference in stent thrombosis (0.37% vs. 0.40%), cardiac death or myocardial infarction (4.5% vs. 5.1%), and target lesion revascularization (3.8% vs. 3.0%) at 1 year between small vessels (2.5 mm stent) and non-small vessels (>2.5 mm stent) (13). Similarly, pooled data analysis from the RESOLUTE global clinical program showed comparable 2-year clinical outcomes between small vessels (reference vessel diameter ≤2.5 mm) and non-small vessels (>2.5 mm) treated with Resolute zotarolimus-eluting stents (14). There was no significant difference in TLF rates between small vessels (10.1%) and non-small vessels (8.7%) at 2 years. The Orsiro stent, used in this study, has ultra-thin struts (60 μm for stent diameter ≤3.0 mm), therefore, has demonstrated better performance compared to other newer generation stents. An analysis of the BIOTRONIK—A Prospective Randomized Multicenter Study to Assess the Safety and Effectiveness of the Orsiro Sirolimus Eluting Coronary Stent System in the Treatment of Subjects With up to Three De Novo or Restenotic Coronary Artery Lesions (BIOFLOW) trials assessed the safety and effectiveness of the Orsiro stent system. It showed a significantly lower incidence of TLF (8.0% vs. 12.4%) and target vessel myocardial infarction (4.2% vs. 7.6%) in patients with small vessel disease (reference vessel diameter ≤2.75 mm) compared to everolimus-eluting stents (15). However, small vessel disease remains an important risk factor for ischemic complications even with the use of this ultra-thin strut stent, as demonstrated in our analysis.

Small coronary arteries have limited capacity to accommodate neointimal growth gollowing stent implantation, leading to a greater reduction in lumen diameter relative to the amount of neointimal thickening, compared to larger vessels (3, 7). Therefore, efforts should be made to achieve a larger stent diameter during PCI through the use of intracoronary imaging (16). It is also important to emphasize the significance of aggressive medical therapy, including the use of potent antiplatelet agents, in high risk patients such as small vessel disease. While potent antiplatelet therapy has shown a reduction in ischemic complications after PCI compared to the conventional P2Y12 inhibitor clopidogrel, direct comparisons in the context of high-risk features such as small vessel disease have been limited (17, 18). In our results, there are no significant interactions between the antiplatelet strategy regarding the 12-month incidence of ischemic complications. Although these results may be due to uncontrolled variables, it suggests that antiplatelet regimen itself may not play a critical role in prevention of TLF in patients under potent P2Y12 inhibitor such as ticagrelor.

To mitigate bleeding complications during DAPT, several studies have explored strategies to shorten the duration of DAPT. Among these studies, the TICO trial investigated a regimen where aspirin was discontinued while ticagrelor monotherapy continued in patients with acute coronary syndrome (4). The results demonstrated that ticagrelor monotherapy administered after 3-month of DAPT was significantly associated with a reduced risk of major bleeding without increasing the risk of major adverse cardio-cerebrovascular events compared to ticagrelor-based 12-month DAPT. This benefit was particularly observed in specific high-risk patient groups, such as those with ST-segment elevation myocardial infarction, diabetes, elderly patients, and obesity (19–22). Our study also demonstrated that ticagrelor monotherapy was not significantly associated with an increased incidence of ischemic events, including TLF and stent thrombosis, and it was not associated with and elevated bleeding risk in patients with small vessel disease. Several pieces of evidence have shown that aspirin does not provide additional platelet aggregation inhibition. In healthy volunteers, potent P2Y12 inhibitors reduce ADP and thromboxane A2 -mediated platelet aggregation, and this effect is minimally enhanced by aspirin (23). In post-PCI patients, ticagrelor monotherapy provided a similar platelet aggregation response to thrombin and thromboxane A2 receptor agonists compared to dual therapy with ticagrelor plus aspirin (24). Therefore, theoretically, ticagrelor could be employed as a single antiplatelet therapy following stent implantation.

This study has several limitations. First, it represents a sub-analysis and was not specifically designed to evaluate the performance of ticagrelor monotherapy in patients with small vessel disease. Therefore, our result should not be interpreted as superior to ticagrelor monotherapy compared to DAPT in patients with small vessel disease. Rather, it should be understood that aspirin withdrawal might be feasible under ticagrelor therapy in small vessel disease. Second, due to limitations in the study design, the sample size is not sufficient to draw definitive conclusions. Future investigations with larger sample sizes and a more rigorous study design are warranted. Third, the use of intracoronary imaging was not investigated in this study, as the original TICO study focused on comparing antiplatelet strategies. Lastly, caution should be exercised when applying the results of this study to non-Asian populations or patients who have received drug-eluting stents other than Orsiro stents, as the findings may not directly generalizable to these populations or devices.

In conclusion, our findings suggest that ticagrelor monotherapy, when compared with ticagrelor-based 12-month DAPT, can effectively reduce bleeding complications without increasing the risk of ischemic events in patients with small vessel disease after the 3-month DAPT period. Further research is warranted to validate and expand upon these findings in larger and more diverse patient populations.

## Data Availability

The original contributions presented in the study are included in the article, further inquiries can be directed to the corresponding author.
